# The learning curve of one anastomosis gastric bypass and its impact as a preceding procedure to Roux-en Y gastric bypass: initial experience of one hundred and five consecutive cases

**DOI:** 10.1186/s12893-020-00697-9

**Published:** 2020-02-26

**Authors:** Hung-Chieh Lo

**Affiliations:** 1 Division of Trauma and Emergency Surgery, Department of Surgery, Wan Fang Hospital, Taipei Medical University, No. 111, Sec. 3, Xinglong Rd., Wenshan Dist., Taipei City, 116 Taiwan, Republic of China; 2 School of Medicine, College of Medicine, Taipei Medical University, No. 111, Sec. 3, Xinglong Rd., Wenshan Dist., Taipei City, 116 Taiwan, Republic of China

**Keywords:** Obesity, One anastomosis gastric bypass, Roux-en Y gastric bypass

## Abstract

**Background:**

The aim of this study was to assess the learning curve of one anastomosis gastric bypass (OAGB-MGB) at the start of a low volume bariatric unit and analyze its impact as a preceding procedure to Roux-en Y gastric bypass (RYGB).

**Methods:**

From January 2014 to December 2017, all patients who underwent bariatric surgeries in our teaching hospital that were performed by the same surgeon were enrolled. The first 47 patients who underwent OAGB-MGB were assigned to group A. RYGB has been offered as a treatment option since July 2016; thereafter, 26 patients who underwent OAGB-MGB and 32 patients who underwent RYGB at the same time interval were assigned to group B and group C, respectively. Baseline characteristics, perioperative outcomes and percentage of total weight loss (%TWL) up to 12 months postoperatively were collected and analyzed between groups.

**Results:**

Compared to the patients in group C, those in groups A and B were older (39.4 yrs. and 42.2 yrs., respectively, vs. 34.2 yrs.; *p* = 0.021) and predominantly male (48.9 and 73.1%, respectively vs. 40.6%; *p* = 0.04), and they had a higher body mass index (41.8 kg/m^2^ and 43.3 kg/m^2^, respectively vs. 37.7 kg/m^2^; *p* = 0.002) and a higher incidence of hypertension (44.7 and 61.5%, respectively vs. 21.9%; *p* = 0.008). In addition, the operation time was significantly reduced (118.2 min and 115.8 min, respectively vs. 153.1 min; *p* <  0.001), and the length of stay was shortened (3.0 days and 2.9 days, respectively vs. 3.4 days; *p* = 0.002) in groups B and C compared to group A. No mortality, conversion or leakage was reported throughout the study period. The 30-day complication rate was decreased in group C compared to groups A and B (0% vs. 6.4 and 7.7%, respectively; *p* = 0.307). The %TWL at the 12-month follow-up was 36.3, 30.9 and 28.3% for groups A, B and C, respectively (*p* <  0.001).

**Conclusion:**

Our study verified the early emergence of a learning curve effect for OAGB-MGB, and the proficiency acquired can be transferred to subsequent practice for RYGB in terms of acceptable operation time and length of stay without an increase in complications.

## Background

Obesity accompanied by multiple comorbidities has become a major global health threat that endangers both quality of life and lifespan [1]. Asia is not exempt from this worldwide trend [2]. While diet, exercise and conventional medical treatment are generally considered ineffective for those with severe or morbidly obese [3], bariatric surgery has been widely accepted as the treatment of choice, with proven sustained long-term efficacy for weight reduction and comorbidity resolution [4]. As a standard procedure, Roux-en Y gastric bypass (RYGB) is technically challenging and takes a steep learning curve to achieve proficiency. Despite the various training courses, mastery classes, proctoring programs, etc., that are currently available to provide assistance [5], the learning process still requires vast cumulative numbers and essential mentoring to maintain a low complication rate. As the relationship between hospital volume and outcomes is well recognized, at least 100 cases annually per hospital is recommended as the minimal requirement to achieve a low risk for serious complications [6]. Moreover, a total experience of 500 cases was deemed necessary to diminish the risk for adverse outcomes and meet safety standards [7]. Although experience in general laparoscopic surgeries may shorten the learning curve for RYGB and improve the safety profile [8], the impact of antecedent skillfulness acquired by other preceding bariatric procedures is rarely reported [9].

On the other hand, despite uncertainties and skepticism, one anastomosis gastric bypass (OAGB-MGB) is being performed by an increasing number of surgeons worldwide and is gradually becoming accepted as a simpler and safer alternative to RYGB with at least equal mid-term efficacy [10–14]. It is also endorsed by the International Federation for the Surgery of Obesity and Metabolic Disorders (IFSO) as a standard treatment option and is no longer considered an investigational procedure [15]. OAGB-MGB comprises all the major steps that are involved in RYGB (stapling, suturing, etc.) except for one less anastomosis (jejunojejunostomy) and lower placed gastrojejunostomy, which obviate the need for omentum division and make suturing more accessible and less cumbersome. In addition, the Petersons’s defect may not need to be closed [16]; these factors are attributed to the perceived ease of implementation. Based on these findings, we felt there was sufficient ground to adopt OAGB-MGB at the beginning of our practice. However, the impact of the accumulated experience gained from performing OAGB-MGB as a preceding procedure to RYGB has rarely been addressed. Being short of prior bariatric experience, our aim was to determine the learning curve of OAGB-MGB from a low-volume unit under careful monitoring and investigate whether proficiency obtained from OAGB-MGB can be transferred to subsequent RYGB with a focus on perioperative outcomes as well as one-year weight loss.

## Methods

We carried out a retrospective analysis from our prospectively maintained database. This study was approved by the local institutional review board, and all procedures performed in this study were in accordance with the ethical standards in the 1964 Declaration of Helsinki and its later amendments. Informed consent was waived because no data regarding the cases were disclosed.

All consecutive patients underwent bariatric surgeries between January 2014 and December 2017 under the care of a single surgeon. Patients were eligible if they had a body mass index (BMI) > 37.5 kg/m^2^ or > 32.5 kg/m^2^ with at least one obesity-related comorbidity, such as type 2 diabetes mellitus (DM), hypertension (HTN), dyslipidemia or sleep apnea. Patients with a BMI < 32.5 kg/m^2^ or those who received operations other than primary OAGB-MGB or RYGB were excluded. Regarding procedure selection, we would like to state that the initial procedures were chosen carefully and based on available evidence at the time. OAGB-MGB served as our preferred procedure because of evidence including a less steep learning curve, shorter operation time, fewer sites for anastomosis and leakage, lower incidence of internal herniation, potential for easier reverse or revision and at least equivalent efficacy in terms of weight loss and comorbidity resolution [10, 11, 14, 17]. We then decided to modify our practice because arguments emerged regarding proper limb length and controversies arose through discussion, such as the long-term consequences of bile reflux and nutrition problems [13, 18, 19]. First, younger patients with lower BMI and those with gastroesophageal reflux disease (GERD) or DM were preferably recommended to receive RYGB starting in July 2016. Second, we modified our OAGB-MGB limb length to a more conservative manner in an attempt to reduce potential long-term nutrition problems. Final selection was made upon a shared decision-making process after fully elaborating the benefits, risks and potential long-term outcomes of each procedure, and all patients were allowed to choose their preferred operation according to surgical consent. Patients were divided into 3 subgroups based on case sequence: group A included the initial patients operated on by the team who underwent OAGB-MGB. The other 2 groups included patients who underwent subsequent surgery. During the second period, those who underwent OAGB-MGB were allocated to group B, while group C comprised the initial patients with RYGB. All patients underwent complete preoperative evaluation, including esophagogastric endoscopy, abdominal sonography, echocardiography, nutritional and endocrinological surveys. Demographic and anthropometric data together with all relevant outcome measures, including the operation time, hospital stay, overall complications and percentage of weight loss up to 12 months postoperatively, were collected and compared between groups A and B to determine the learning curve of OAGB-MGB and between groups A/B and C to study the impact of preceding OAGB-MGB on subsequent practice of RYGB. A BMI of 25 kg/m^2^ was set as the ideal [20]. Postoperative complications were classified as major (CD > IIIa) or minor based on the Clavien-Dindo classification (CD) [21] and as early or late by the time of onset (early, < 30 days; late, > 30 days). Nutrition supplementation and yearly surveys, including iron, Vit. B12, folate, calcium and vitamin D were carried out for all our patients.

### Surgical technique

RYGB was performed by constructing a 30-mL vertical gastric pouch over a 32 Fr. calibrating tube, followed by an average 100 cm ante-colic alimentary, 100 cm biliary limb; linear stapled gastrojejunostomy, jejuno-jejunostomy with enterotomy closed with absorbable sutures. Mesenteric defects were routinely closed via nonabsorbable sutures.

For OAGB-MGB, the technique involves first stapling via Crow’s foot and subsequent multiple firings alongside a Fr. 32 calibration tube with stapled gastrojejunostomy and closure of enterotomy with absorbable sutures. Biliopancreatic limb length varied according to BMI, although consensus has not yet been reached. At first, the strategy was with a 5 cm increment from a baseline length of 180 cm for every BMI category increase above 40 until a maximum length of 300 cm was reached. The limb length was measured under the aid of a 5 cm suture line as a reference to minimize errors. We then modified and converted our limb length into a range from 180 cm to 200 cm during the second period. The average biliopancreatic limb length was 247 cm in group A and 192 cm in group B. All patients were regularly followed up at 1, 3, 6, and 12 months postoperatively and annually thereafter.

## Data collection and statistical analysis

The Statistical Package for the Social Sciences software version 20.0 (SPSS Inc., Chicago, Illinois, USA) was used for statistical analyses. Continuous variables were reported as the means ± standard deviations. Categorical variables were expressed as counts and percentages. Chi-square tests or Fisher’s exact tests were used to compare two categorical variables. One-way ANOVA was used to detect differences in continuous variables among the three groups. Tests for statistical significance were two-sided with a level of significance of 0.05.

## Results

Between January 2014 and December 2017, a total of one hundred and twenty patients underwent bariatric surgeries in our hospital. Among them, 10 patients with BMI < 32.5 kg/m^2^, three patients who underwent sleeve gastrectomy (SG) and another two patients who underwent nonprimary surgeries were excluded, leaving one hundred and five patients enrolled in this study. Of these, the first 47 patients who underwent OAGB-MGB were assigned to group A; from July 2016, 26 patients who underwent OAGB-MGB and 32 patients who underwent RYGB at the same time interval were as assigned to group B and group C, respectively.

Demographic data and the clinical characteristics of the patients are reported in Table 1.

**Table 1:** Demographic and clinical characteristics of patients, mean (SD)

	OAGB Group A (*N* = 47)	OAGB Group B (*N* = 26)	RYGB Group C (*N* = 32)	*p*-value
Age (years)	39.4 ± 11.3	42.2 ± 12.7	34.2 ± 9.4	0.021
Sex, n (%)				0.040
Male	23 (48.9)	19 (73.1)	13 (40.6)	
Female	24 (51.1)	7 (26.9)	19 (59.4)	
Preoperative weight (kg)	116.1 ± 23.9	127.3 ± 18.4	104.8 ± 15.9	< 0.001
BMI (kg/m^2^)	41.8 ± 7.6	43.3 ± 5.8	37.7 ± 3.1	0.002
Comorbidities, *n* (%)	38 (80.9)	23 (88.5)	24 (75.0)	0.430
Diabetes mellitus, *n* (%)	14 (29.8)	13 (50.0)	9 (28.1)	0.149
Hypertension, *n* (%)	21 (44.7)	16 (61.5)	7 (21.9)	0.008
Dyslipidemia, *n* (%)	25 (53.2)	16 (61.5)	19 (59.4)	0.752
GERD, *n* (%)	9(19.1)	8(30.8)	15(46.9)	0.032

Data are expressed as the mean ± standard deviation

*OAGB* one anastomosis gastric bypass, *RYGB* Roux-en Y gastric bypass, *BMI* body mass index, *GERD* gastroesophageal reflux disease

Patients in group B were older than those in group A or C (42.2 yrs., 39.4 yrs. and 34.2 yrs., respectively; *p* = 0.021) and predominantly male (73.1, 48.9 and 40.6%, respectively; *p* = 0.04). Group B also had the highest baseline BMI, with group A in between, while group C had the lowest BMI (43.3 ± 5.8 kg/m^2^, 41.8 ± 7.6 kg/m^2^ and 37.7 ± 3.1 kg/m^2^, respectively; *p* = 0.002). The incidence of comorbidities was not different among the three groups (88.5, 80.9 and 75%, respectively; *p* = 0.43). However, group B had a tendency toward more cases of DM (50, 29.8 and 28.1%, respectively; *p* = 0.149) and had significantly more cases of HTN (61.5, 44.7 and 21.9%, respectively; *p* = 0.008) compared with groups A and C. In regard to the preoperative incidence of GERD, group C consisted of significantly more patients with GERD grade A, B to group B and Group A (46.9, 30.8 and 19.1%, respectively; *p* = 0.032). None of our patients had GERD grades equal to or greater than grade C. Only two patients in group B had small hiatal hernias and hence were not repaired.

As shown in Table 2, there was a significant decrease in the operation time between group B and group A (118.2 min vs. 153.1 min, respectively), which reached a steady state in group C (115.8 min). The mean hospital stay was 3.4 days, 3 days and 2.9 days in groups A, B and C, respectively (*p* = 0.002). All procedures were complete by laparoscopic approach without conversion to open surgery.

**Table 2:** Surgical perspectives and outcomes, mean (SD)

	Group A (*N* = 47)	Group B (*N* = 26)	Group C (*N* = 32)	*p*-value
Op time (min)	153.1 ± 42.2	118.2 ± 23.5	115.8 ± 30.5	< 0.001
LOS (days)	3.4 ± 0.8	3.0 ± 0.3	2.9 ± 0.2	0.002
Early complications, *n* (%)	3 (6.4)	2 (7.7)	0 (0.0)	0.307
Intra-abdominal hematoma	1	0	0	
Melena	0	1	0	
Nausea/vomiting	1	0	0	
Operation abandoned^a^	0	1	0	
Stapling of calibration tube	1	0	0	
Readmission	2	0	0	
Late complications, *n* (%)	8(17.0)	3(11.5)	2 (6.3)	0.357
Marginal ulcer	4	2	2	
G-J stenosis	1	0	0	
anemia	2	1	0	
malnutrition	1	0	0	
Mortality, *n* (%)	0	0	0	

*Op* Operation, *LOS* length of stay, *G-J* gastrojejunostomy

^a^ The patient experienced bulky liver intraoperatively and the index operation had to be abandoned

The rate of early complications was higher in groups A and B than in group C (6.4, 7.7 and 0%, respectively; *p* = 0.307), but there was no statistical significance. Of these, a total of 3 patients in group A experienced early complications. Among them, one patient suffered from a left subphrenic hematoma and was readmitted for percutaneous drainage (CD IIIa). A second patient was readmitted on postoperative day (POD) 7 for nausea/vomiting and recovered uneventfully. The third patient experienced stapling of the calibration tube intraoperatively. Notably, one particular patient presented to the emergency room at POD4 with mild fever but did not require readmission or further treatment; therefore, were not classified as having complications by definition.

Two patients suffered from complications within 30 days in group B. One patient experienced a bulky liver intraoperatively, and the index operation had to be aborted. This patient was a 45-year-old male patient with a BMI of 43.4 kg/m^2^ and severe fatty liver. Under strict dietary control, reoperation was performed uneventfully 4 months later. A second patient was noted to have self-limited melena on POD2 and was discharged on POD4. There were no perioperative complications in group C. No leakage or mortality was reported in this study.

The late complication rates were 17.0, 11.5 and 6.3% in groups A, B and C, respectively (*p* = 0.357). According to our follow-up regimen, because there was no severe GERD (grade ≥ C) preoperatively and the presence of clinically significant hiatal hernia was uncommon throughout the study period, esophagogastric endoscopy was indicated postoperatively only for patients with significant symptoms of GERD, suspected ulcer-related problems, stenosis, etc. Consequently, we do not have an overall picture of the interval change in preexisting GERD or the incidence of de novo reflux. The results are four marginal ulcers in group A and 2 marginal ulcers each in group B and group C. Another patient in group A was diagnosed with gastrojejunostomy stenosis at 12 months postoperatively and relieved under a single session of balloon dilatation.

During the one-year follow-up period, two patients in group A had anemia and took additional iron supplements. Each patient had biliopancreatic limb lengths of 280 cm and 240 cm, respectively. In group B, there was only one patient with preexisting anemia who suffered from aggravated anemia postoperatively and needed extra iron replacement. Another patient in group A suffered from malnutrition that occurred at 8 months postoperatively, which require parenteral nutrition. The biliopancreatic limb length was 230 cm for this particular patient. No anemia or malnutrition was reported in group C.

Up to 12 months postoperatively, 81% of the patients in group A, 85% of the patients in group B and 63% of the patients in group C were available for follow-up. The weight loss results are shown in Table 3. Statistically significant differences in terms of percentage of total weight loss (%TWL) and percentage of excess weight loss (%EWL) were found. The %TWL was 36.3, 30.9, and 28.3% in groups A, B and C, respectively (*p* <  0.001). The %EWL was 92.9, 77.2, and 85.5% in groups A, B and C, respectively (*p* = 0.006). Group A had a greater %EWL and %TWL at 12 months postoperatively.

**Table 3:** Twelve month weight loss outcomes

	Group A (*N* = 47)	Group B (*N* = 26)	Group C (*N* = 32)	*p*-value
Patients at follow-up, *n* (%)	38(81)%	22 (85%)	20(63%)	
%EWL, mean ± SD	92.9 ± 21.0	77.2 ± 13.7	85.5 ± 12.7	0.006
%TWL, mean ± SD	36.3 ± 7.8	30.9 ± 5.5	28.3 ± 4.7	< 0.001

*%EWL* percentage of excess weight loss, *%TWL* percentage of total weight loss

## Discussion

Herein, we report the learning curve of a total of 105 patients who underwent surgery over a 4-year period in a low-volume hospital from the inception of a new bariatric project. In particular, we analyzed the impact of performing OAGB-MGB as a preceding procedure to RYGB. Comparing the results among the 3 groups in terms of perioperative and postoperative outcomes as well as one-year weight loss, continued improvement in terms of the operation time and length of stay was found in patients who receive OAGB-MGB across the study period (group B to group A), and this positive impact could be transferred to those who underwent RYGB subsequently (group C), with all three groups showing successful weight loss up to 12 months postoperatively.

RYGB is generally recognized as a difficult operation that includes several technical points the surgeon must master to achieve acceptable morbidities and avoid mortalities. Clearly, to some extent, a sophisticated laparoscopic skill set is a prerequisite before starting with such a complex operation [22]. At least 75–100 cumulative cases are recommended to overcome the learning curve [23, 24], and some believe up to 500 cases are necessary to reach proficiency and stabilize perioperative risk [7, 25]. One systemic review reported that inexperienced surgeons or those without a proper training background were riskier and have substantially higher major complication and mortality rates [5]. In contrast, Keller et al. [9] suggested that it is safe to initiate a new program if there is experience from other preceding bariatric procedures, such as gastric banding. Breaux et al. [8] proposed that bariatric fellowship or mentoring may not be required for cases in which surgeons already have advanced laparoscopic skills. In addition, the surgeon’s personal skills, training background and hospital volume to some degree are all impactful [26]. To summarize, there are no formal certification processes or quality measures with respect to readiness for RYGB.

In contrast, being gradually acknowledged as a feasible choice among mainstream bariatric surgeries [15, 27], OAGB-MGB is generally reported to have lower risk profiles than RYGB [10–13, 17, 28]. In a cohort of more than 1600 patients, Lee et al. [10] concluded that OAGB-MGB has a shorter operation time, lower major complication rate (1.8 vs. 3.2%, *p* = 0.07), and higher excess weight loss than RYGB. With respect to its learning curve, Parmar et al. [29] reported their first 125 OAGB-MGB series that accomplished a mean operation time of just 92.4 min and postoperative hospital stay of 2.2 days with only one early and four late reoperations. Wang et al. [30] reported 423 consecutive patients in their early experience with a major complication rate as low as 1.7%. With an average operation time of 130.8 min, a mean hospital stay of 5 days and a 4.3% early complication rate, they concluded that the learning curve for OAGB-MGB was less steep than that for RYGB. Rutledge R [31]. depicted an overall complication rate of only 5.2% in his first large series, which comprised as many as 1274 cases with a mean operation time of 36.9 min and length of stay of 1.5 days. Therefore, OAGB-MGB was our preferred choice in this work due to the aforementioned factors. However, it is worth noting that it is not our intent to argue against the importance of formal training programs or debate the justifiability of the specific procedure with initial skepticism. Since there are no established guidelines for gauge qualification, we deemed that we were ready for to perform this procedure after attending a short-term dry lab course and live demonstrations; in addition, we have prior experience in various advanced laparoscopic gastrointestinal and hepatobiliary surgeries.

In fact, upon further examination of the first 47 OAGB-MGB patients that comprise group A, it appears that our results are inferior to those reported in the abovementioned studies, with a mean operation time of slightly over two and a half hours, a length of stay of 3.4 days and an early complication rate of 6.4% [29–31]. However, we deemed our initial results to be acceptable because there was no open conversion, leakage or other major complications (CD > IIIa). Furthermore, comparing the results of OAGB-MGB between two time frames, a notably significantly reduced operation time (118.2 min vs. 153.1 min) and shortened length of hospital stay (3 days vs. 3.4 days) were achieved throughout the learning process despite group B comprising higher-risk patients than group A—namely, older and heavier patients who were predominantly male [32]. Our result is consistent with prior reports and verified the early emergence of the learning curve effect [17, 30]. Nonetheless, due to the retrospective design and nonrandomized nature of this study, patient selection bias cannot be eliminated. Part of the reason for this bias can be explained by the fact that younger patients, those with lower BMI, and those with GERD or DM were preferably recommended for RYGB during the second period, even though there was lack of evidence to support this recommendation strategy. Therefore, we used similar recommendations to select lower-risk patients for RYGB during the early phase of practice [33]. Likewise, others have preferentially offered OAGB-MGB over RYGB to patients with perceived technical difficulties [29]. There is evidence to show that OAGB-MGB is safer and offers better weight loss outcomes than RYGB and SG in superobese patients according to a systematic review [34]. With only one planned reoperation in group B due to obscured operation field and early complication rate of 7.7%, we believe that the results of our learning process were appropriate. Additional data regarding its long-term efficacy and safety were followed closely.

On the other hand, implementing RYGB with insufficient surgical experience and limited patient numbers can lead to unfavorable results, with an overall complication rate ranging from 23.3 to 32% [24, 35], a high conversion rate and prolonged operation times over 3 h [35]. In a series of a single surgeon’s work, Oliak D [24]. reported his early experience with 75 consecutive RYGB cases, which took a mean operation time of 189 min and demonstrated a total complication rate of 32%. Notably, there were 10 major complications, 2 conversions and 2 deaths. Keller P [9]. conducted a study in their first 50 RYGB cases with a mean hospital stay of 5.3 days and a total complication rate of 10%. Huang C.K [36]. demonstrated the feasibility and safety of conducting RYGB in a series of one hundred Chinese patients. Notably, he had an average operation time of 216 min and described that the risks were significantly higher in his first 50 cases. The overall complication rate in his series was 18%, while the major complication rate was 8%. In our series, we found continued improvement in terms of the operation time (153.1 min v. 118.2 min vs. 115.8 min; *p* <  0.001) and length of stay (3.4 days vs. 3 days vs. 2.9 days; *p* = 0.002) throughout the study period. In contrast to most studies, which showed a shorter operation time for OAGB-MGB than for RYGB [10, 12, 17, 37], our operation time in group B was slightly longer than that in group C. Different from the aforementioned studies, we lacked vast prior experience, and our study was not based on a dedicated or specialized bariatric unit. In other words, the plateau during the learning process may not yet have been reached after 73 accumulative cases, although we managed to reduce the operation time markedly from group A to group B. Additionally, the early discovery of a high marginal ulcer rate in group A rendered us to adopt a more meticulous approach, and we attempted to construct a narrower gastric pouch in group B. Our results can also be partially explained by differences in the demographic characteristics of the patients in groups B and C, with group C comprising younger patients (34.2 yrs. vs. 42.2 yrs) with a lower BMI (37.7 ± 3.1 kg/m^2^ vs. 43.3 ± 5.8 kg/m^2^), as well as less male predominance (40.6% vs. 73.1%), for these are well-identified factors that affect the operation time. Despite this, the noninferior result obtained in group C compared to group B can still be considered a continued improvement by acquired proficiency. Considering a 3–5% major complication rate during the learning process of the first 100 RYGB cases to be successful [17], our first 32 consecutive RYGB series fulfilled this criterion, as there was no leakage, reoperation or other major complications. With regard to other commonly referred indicators for overcoming the learning curve, such as conversion rates of 1–3% and operation times of < 2 h [36, 38], our results are within this scope because our mean operation time was 115.8 min, and there were no conversions. Our study revealed that it is safe to perform RYGB for lower-risk patients after gaining proper experience via accessible preceding bariatric procedures.

Of these patients, better weight loss was found in terms of %TWL and %EWL in group A (36.3 and 92.9%, respectively) compared with group B (30.9 and 77.2%, respectively) and group C (28.3 and 85.5%, respectively). We think this is because we were more conservative with regard to the biliary limb length for OAGB-MGB during the second period, and OAGB-MGB is commonly reported to have equal to or slightly better results than RYGB in terms of weight loss [10]. The reported %EWL also tends to be confounded by the original body weight. Since group B had the highest mean BMI, part of its lowest %EWL can be explained.

We noticed a high marginal ulcer rate in our series, although we paid more attention to constructing a gastric pouch during the second period and thereafter. Six ulcers were related to smoking, and another was related to the postoperative abuse of nonsteroid anti-inflammatory drugs. Since then, we have tracked the problem more closely and reinforced the importance of quitting smoking and extending the use of a postoperative proton pump inhibitor to 6 months as much as possible. On the other hand, successive cases suffering from anemia and nutritional problems in group A were found with biliopancreatic limb lengths varying from 230 cm to 280 cm, and a more conservative approach by reducing the mean biliopancreatic limb length from 247 cm to 192 cm has since been adopted. More relevant data and closer follow-up are needed to clarify the long-term nutritional impacts and consequences for weight loss or comorbidity resolution for this practice.

### Limitations

The limited patient numbers and short-term follow-up inherent to the study background hinder the interpretation of our study. Notably, there is insufficient statistical power to detect meaningful differences regarding our end-points and the possibility of statistical type II errors. However, we did not attempt to conduct a head-to-head comparison for learning curves or various aspects between each procedure, and it is also far beyond the scope for in-depth discussion of individual indications or contraindications. Furthermore, a higher one-year attrition rate in group C compared to group A and group B brings further bias and limits the generalization of our results. That being said, as the aim of this study was to evaluate the initial safety and feasibility of conducting a new bariatric project step-by-step from a low-volume unit, which was verified. The collection of more long-term data and more relevant data, such as the resolution of comorbidities, quality of life, nutrition assessment, and reoperation/revision, requires more solid auditory and follow-up programs and could provide more robust evidence to support successful results early in the learning curve and help continue to refine our practice.

## Conclusion

In summary, a less steep learning curve was verified for OAGB-MGB, with continued positive influence that can be transferred to subsequent practice for RYGB. However, the potential for attrition bias while interpreting the reported outcome measures should be considered. For this reason, in the pursuit of identifying the beneficial effects of bariatric surgery, we should conduct our bariatric project more cautiously in the future and continue to monitor relevant safety profiles relentlessly.

## Data Availability

The data that support the findings of this study are available from the Taipei Medical University Joint Institutional Review Board, but restrictions apply to the availability of these data, which were used under license for the current study and are not publicly available. Data are, however, available from the authors upon reasonable request and with permission from the Taipei Medical University Joint Institutional Review Board.

## Abbreviations

%EWL: Percentage of excess weight loss
BMI: Body mass index
CD: Clavien-Dindo classification
DM: Diabetes mellitus
GERD: Gastroesophageal reflux disease
HTN: Hypertension
OAGB: One anastomosis gastric bypass
POD: Postoperative day
RYGB: Roux-en Y gastric bypass %TWL Percentage of total weight loss
